# Willingness of Chinese nurses to practice in Hubei combating the coronavirus disease 2019 epidemic: A cross‐sectional study

**DOI:** 10.1111/jan.14434

**Published:** 2020-06-22

**Authors:** Xiaoqing Gan, Zeya Shi, Sek Ying Chair, Xi Cao, Qun Wang

**Affiliations:** ^1^ Hunan Provincial People's Hospital Changsha China; ^2^ The Nethersole School of Nursing The Chinese University of Hong Kong New Territories Hong Kong; ^3^ School of Nursing Shenzhen University, Nanshan District Shenzhen China

**Keywords:** COVID‐19, epidemic, infectious disease, nurses, staffing, willingness

## Abstract

**Aims:**

To investigate the willingness of Chinese nurses to practice in Hubei combating the coronavirus disease 2019 and to explore the associated factors.

**Design:**

A cross‐sectional survey.

**Methods:**

Clinical nurses were conveniently recruited by an online link in three provinces out of Hubei, including Hunan (Central south), Chongqing (Southwest) and Xinjiang (Northwest) during 4–10 February 2020. A structured questionnaire was distributed by an online investigation system. Information on sociodemographic characteristics, willingness, possible influencing factors (previous experience, health status, training conditions, perceptions on volunteering to practice in Hubei, family attitude, and insurance) was collected. Binary logistic regression was conducted to explore the association of different factors with the willingness decision of nurses.

**Results:**

A total of 11,183 nurses participated in this survey and a high proportion of them were willing to volunteer to practice in Hubei combating the epidemic. Nurses who were likely to volunteer had the following characteristics: younger, unmarried, members of the Communist Party of China, with senior professional qualification, working in critical care departments, with support from their families, with adequate training and learning, with good health status and low levels of anxiety. The regression model could explain 31.1% of the variances of the willingness decision of nurses.

**Conclusions:**

A high proportion of nurses in China were willing to practice in Hubei during the coronavirus disease 2019 epidemic. Adequate training and psychological support would facilitate nurses to volunteer during the outbreak of an infectious disease.

**Impact:**

The study identified a high proportion of nurses in China were willing to practice in Hubei combating the coronavirus disease 2019 epidemic. The findings will provide valuable references for nurses and decision makers to formulate better plans for increasing nursing workforce during such kind of public health crisis.

## Abstract (zh)

**目的:**

本研究旨在调查中国护士赴湖北抗击2019年新冠肺炎病毒疫情的执业意愿,并探讨相关因素。

**设计:**

一项横断面研究。

**方法:**

2020年2月4日至2020年2月10日期间,通过网络链接,迅速招募到湖北以外的湖南(中南部)、重庆(西南部)、新疆(西北部)这3个省份的临床护士人员。通过网络调查系统分发结构式问卷。收集了社会人口特征、意愿、可能的影响因素(以往经历、健康状况、培训条件、对前往湖北进行志愿服务的看法、家属态度、保险情况)等信息。进行二元logistic回归分析,探讨不同因素与护士意愿决策的关联。

**结果:**

共有11183名护士参与了此次研究,其中有很大一部分护士愿意赴湖北提供志愿服务,抗击疫情。有意愿成为志愿者的护士具有以下特征:年轻、未婚、中共党员、具有高级职业资格、在重症科室工作、有家人支持、接受过适当培训和学习、健康状况良好、焦虑程度低。回归模型显示了31.1%的护士意愿决策差异。

**结论:**

2019年新冠肺炎病毒流行期间,中国有很大一部分护士愿意赴湖北开展相关工作。适当的培训和心理支持将促进护士在传染病爆发期间自愿提供服务。

**影响:**

研究发现,中国有很大一部分护士愿意赴湖北抗击2019年新冠肺炎病毒疫情。研究结果为护士和决策者提供了有价值的参考,针对此类公共卫生危机事件,护士和决策者可据此制定更适宜的护理队伍扩增方案。

## 1 INTRODUCTION

An ongoing outbreak of a respiratory disease caused by the 2019 novel coronavirus (COVID‐19) emerged in Wuhan City, Hubei Province, China in December 2019 (The World Health Organization [WHO], 2020). This new virus has a similar genome sequence with the Severe Acute Respiratory Syndrome‐Associated Coronavirus (SARS‐CoV) but is less pathogenic than the SARS‐CoV and the Middle East Respiratory Syndrome coronavirus (MERS‐CoV) (Chen, 2020). The COVID‐19 has a strong capability of human‐to‐human transmission, resulting in its rapid spread throughout China and later outside the country (Cao et al., 2020). Until 24 February 2020, the number of confirmed cases in Mainland China had grown to 77,658, including 2,663 deaths and 47,672 under medical treatment; among these cases, 64,786 diagnosed patients and 2,563 deaths occurred in Hubei, the epicentre of the outbreak (National Health Commission of the People's Republic of China [NHC], 2020a). In response to the COVID‐19 outbreak, China has activated various emergency responses, such as holiday extensions, building specialty hospitals, the lockdown of Wuhan, a nationwide quarantine policy and sending a huge number of healthcare professionals from other parts of the country to help Hubei (Foreign Policy, 2020, NHC, 2020b).

Nurses, the professionals with the most contact with patients, play an essential role in fighting against disease outbreaks. In addition to initiating nursing procedures, the outbreak of an epidemic imposes frontline nurses with additional roles, such as screening and recognizing potential cases, helping with isolation, implementing a quarantine and monitoring cases (Stirling, Hacher, & Harmston, 2017). Therefore, a substantial increase of nursing workforce is demanded to assume these responsibilities. The nurse‐to‐population ratio in Hubei is 2.65 per 1,000 people (National Bureau of Statistics, 2019), lower than the average level in Mainland China (3.14) (CNKI, 2020) and much lower than that in Japan (11.5) and the USA (8.6) (The World Bank, 2019). The 47,672 patients with COVID‐19 require intensive treatment and care (NHC, 2020a), thus imposing additional burden to the inadequate nursing workforce there. Caring for this large number of patients places substantial stress on local nurses and may lead to their burnout.

To control this outbreak and to relieve the healthcare workforce shortage at the frontlines, physicians, nurses and other healthcare professionals have been recruited to practice in Hubei combating this epidemic. Till 17 March 2020, over 42,600 healthcare professionals from different hospitals all over the country, including 28,600 nurses, were sent to Hubei to fight against COVID‐19 epidemic (National Health Commission of the People’s Republic of China, 2020c). These healthcare professionals that were sent to Hubei were organized by local health commissions (the government department). Therefore, there is a need to understand the willingness of nurses to practice in Hubei and the factors associated with their willingness.

### 1.1 Background

Engaging healthcare professionals to serve during infectious disease outbreaks remains a difficult management topic. Previous studies reported a high proportion of absenteeism in Hong Kong (76.9%) (Wong et al., 2010), Taiwan (43%) (Lee et al., 2005), Australia (33%) (Stuart & Gillespie, 2007) and Germany (28%) (Ehrenstein, Hanses, & Salzberger, 2006) during the influenza pandemic, outbreaks of SARS and other infectious diseases. A survey in UK reported that only 1.7% of healthcare professionals volunteered to work in West Africa for the Ebola epidemic (Turtle et al., 2015).

The willingness of healthcare professionals to serve at the frontline of an outbreak is influenced by various factors. It may also vary from epidemic to epidemic and from region to region. Psychological stress, previous experience with an epidemic, safety concerns on being infected, social support, and the attitudes of the families were associated with willingness of healthcare professionals to serve in the frontline (Khalid, Khalid, Qabajah, Barnard, & Qushmaq, 2016; Oh et al., 2017; Turtle et al., 2015). Specifically, the common facilitators for healthcare professionals being volunteers include receiving training, availability of effective treatments (Turtle et al., 2015), perceived professional obligation, support from the hospital administration, financial compensation (Khalid et al., 2016), adequate protective equipment, reasonable staffing, and family support (Lee et al., 2005). Insufficient information, worry of being infected and the concerns of their families are the most cited reasons for nurses to not help in the frontline (Turtle et al., 2015).

With the strong transmission of COVID‐19 and the rapid increasing number of patients, an escalating demand for nursing workforce exists in Hubei, the epicentre of this outbreak. Nurses’ willingness to help fighting against COVID‐19 is a critical issue in the nursing management. Therefore, the current study was conducted to identify the willingness of Chinese nurses to practice in Hubei combating COVID‐19 and to explore the associated factors. Findings from this study will provide valuable references for decision makers to formulate better plans for increasing nursing workforce during such kind of public health crisis.

## 2 THE STUDY

### 2.1 Aims

The study aimed to investigate the willingness of Chinese nurses to practice in Hubei combating the coronavirus disease 2019 (COVID‐19) and to explore the associated factors.

### 2.2 2 Design

This study was a cross‐sectional study. Given the strong capability of human‐to‐human transmission of the COVID‐19, close contact and mass gatherings should be limited (WHO, 2020). Considering the wide use of social media in China, for example, WeChat and QQ, an online survey approach was adopted, taking advantage of high efficiency and low cost.

### 2.3 Study settings

The online survey was conducted in the rising stage of this epidemic from 4–10 February 2020 shortly before the peak. The peak of the COVID‐19 epidemic in China occurred on 12 February, with 15,152 new cases and 254 new deaths on that day (NHC, 2020d). The survey was distributed in three province‐level administrative divisions in China, including Hunan Province (Central South China), Chongqing City (a province‐level municipality directly under the Chinese central government, Southwest) and Xinjiang Uygur Autonomous Region (a province level administrative division, with a main ethnic group of Uygur, Northwest). The three province‐level administrative divisions are located in different areas (North and South, Central and West) in China, with diverse ethnic compositions, populations, gross domestic product levels, cultural backgrounds and medical resources. Moreover, Hunan and Chongqing are both adjacent to Hubei, while Xinjiang is over 3,000 kilometres northwest of Hubei. Recruiting participants from the three areas would provide a full picture of nurses with various characteristics, especially with the differences in geographic location and distance from the epicentre of the outbreak.

#### 2.3.1 Participants

Registered Nurses working in clinical practice were invited.

According to the most updated statistics, the total number of registered nurses was 184,000, 95,100 and 72,300 in Hunan, Chongqing and Xinjiang, respectively (National Bureau of Statistics, 2019). A convenient sample of at least 1% of the total number of nurses in each province was expected in the current study (Sun & Xu, 2014). Accordingly, at least 3,514 nurses were required, including 1,840, 951 and 723 in Hunan, Chongqing and Xinjiang, respectively.

#### 2.3.2 Data collection

##### Study instrument

Based on findings of previous studies (Khalid et al., 2016; Lee et al., 2005; Oh et al., 2017; Turtle et al., 2015) and a pilot test, a structured questionnaire was developed to investigate Nurses’ Willingness of volunteering to practice in Hubei and the Influencing Factors (NWIF). The NWIF included 27 items, covering the sociodemographic characteristics (10 items), willingness of volunteering to help Hubei (1 item), and influencing factors (16 items). The sociodemographic characteristics included age, gender, education, political party membership, professional qualification, working department, marital status, number of children, living conditions, and locations.

The willingness was asked by the item ‘would you like to be a volunteer for Hubei to combat the COVID‐19 epidemic?’. A volunteer means the nurse would leave his or her original working hospital and hometown and practice in a hospital in Hubei to fight against COVID‐19. All volunteers to Hubei were selected and organized by local health commissions. When the job in Hubei is completed, the volunteers will return to their original working positions.

The possible influencing factors included the aspects of personal health (three items), previous experience (four items), training condition (three items), personal perceptions (four items), family attitude (one item) and insurance (one item). The perceived health status was measured by the question, ‘How do you feel about your current health status?’, with the choices of ‘energetic and spirited, as stable as before, feel depressed, unhappy for a long time, sleep disorders and others’. The perceived stress and anxiety levels were measured by the visual analogy scales (VAS), with 0 indicating no stress/anxiety and 100 indicating the highest level of stress/anxiety. The related experience items included ‘whether you have participated in a similar kind of public health crisis’, ‘whether your friends/colleagues have participated in a similar kind of public health crisis’, ‘whether you had close contact with COVID‐19 patients’, ‘whether any colleague was isolated’, ‘whether you bought a specific insurance for this crisis’. The training conditions included ‘whether you have received training for COVID‐19’, ‘your understanding about COVID‐19‐related knowledge’ (with choices of ‘very good/good/partly/not understanding’) and ‘how much time per day you spent on learning COVID‐19‐related knowledge’. The perceptions of nurses on the greatest benefits and worst outcomes of volunteering to help Hubei, the facilitators and barriers for the willingness decision were also asked. The attitudes of families towards being a volunteer were asked by one question, with the choices of ‘strongly support, support, a bit not support and not support at all’. The information on whether bought insurance for oneself was also asked by one item with yes‐or‐no choices.

### 2.4 Procedures

The questionnaire was presented in the Wenjuanxing online investigation system (www.wjx.cn) with a unique link. The researchers sent the link and introduction of the study to possible nurse administrators of different hospitals in the study areas. The survey link was thereby disseminated to clinical nurses through WeChat groups and QQ groups. The participation of this study was voluntary as there was no penalty nor award offered. The nurses could click the link and launch the first page, which introduced the aims, study criteria and process of the survey. The contact information of the investigators was also provided. At the end of the first page, the question: ‘do you agree to participate in this survey’ was asked to acquire the consent of the nurses. Only the ‘yes’ option led to the next page for the questionnaires. One mobile IP could only submit the answers once, which prevented duplication. No identity information was collected. The participants were only identified by the sequence numbers generated by the Wenjuanxing system. The online survey was discontinued a week later when the sample size was satisfied.

### 2.5 Ethical considerations

The current study followed the Declaration of Helsinki. The study was approved by the Ethical Committee of Hunan Province People Hospital (No. 2020004).

### 2.6 Data Analysis

The data were exported from the online investigation system. SPSS software was used for data analysis. The statistics of mean, standard deviation (*SD*), count and percentage were employed to describe the characteristics and responses of the participants. The characteristics of participants who were willing or not to volunteer were presented. Chi‐square tests and *t‐*tests were used to explore the differences among the proportions of willingness among nurses with different characteristics. Binary logistic regression was conducted to explore the association of different factors with the willingness decisions of nurses. The willingness of nurses was analysed as the dependent variable (‘willing’ =1, ‘not willing’ = 0). Possible associated factors were explored in the ‘enter’ method. Odds ratio (OR) and the 95% confidence intervals (CI) were computed. A *p* value less than .05 was set as statistical significance.

### 2.7 Validity and reliability

The NWIF was developed by the research team based on literature review (Khalid et al., 2016; Lee et al., 2005; Oh et al., 2017; Turtle et al., 2015). The content validity of the questionnaire was evaluated by an expert panel, including two clinical nurses, a nurse manager, a hospital administrator and two nursing professors. A pilot test was conducted among 50 nurses in a hospital in Hunan Province to examine the readability, clarity and coverage of the questionnaire. Revisions were made based on nurses’ comments in the pilot test. The content validity index was 0.92 for NWIF. The reliability (Cronbach's a) of NWIF was 0.71 in the current survey.

## 3 RESULTS

A total of 11,283 responses were received, among which 100 responded ‘No’ in the consent question. Finally, 11,183 responses were analysed, with a response rate of 99.1%. Among the participants, 2,342 (20.9%) were from Hunan, accounting for 1.27% of local nurses; 5,758 (51.5%) were from Chongqing, accounting for 6.05% of local nurses; and 3,121 (27.6%) were from Xinjiang, accounting for 4.32% of local nurses. The 1‐week online survey fully met the minimum sample size requirement, indicating the feasibility and high efficiency of this approach.

### 3.1 Demographic characters

The characteristics of the study participants were presented in Table 1. Most participants in the current study were female nurses (96.7%), aged below 40 years (89.2%), with no political party membership (87.7%), with bachelor's degree or above (56.5%), married (69.3%), with at least one child (61.5%), and living with their parents (53.1%). Most nurses had primary (73.8%) or middle (21.9%) levels of professional qualification and had more than 5 years of working experience (66.8%). In the current study, 21.6% of the nurses worked in high‐risk departments for contacting with COVID‐19 (NHC, 2020e), for example, critical care departments (11.7%), emergency department (6.3%) and infectious disease departments (3.6%).

**Table 1:** The characteristics of the nurses (*N* = 11,183)

Variables		*N*	(%)
Sociodemographic characteristics			
Location	Hunan	2,342	(20.9)
	Xinjiang	3,083	(27.6)
	Chongqing	5,758	(51.5)
Gender	Male	372	(3.3)
	Female	10,811	(96.7)
Age	20–29 years	5,516	(49.3)
	30–39 years	4,464	(39.9)
	≧40 years	1,203	(10.8)
Professional qualification	Primary (registered nurses)	8,258	(73.8)
	Middle (nurses in charge)	2,448	(21.9)
	Senior (chief nurses)	477	(4.3)
Political party membership	No	9,809	(87.7)
	The Communist Party of China	1,260	(11.3)
	Other democratic parties	114	(1.0)
Education	Associate degree	4,863	(43.5)
	Bachelor degree	6,224	(55.7)
	Master degree or above	96	(0.8)
Working departments	Emergency department	706	(6.3)
	Infectious departments	405	(3.6)
	Critical care units	1,306	(11.7)
	Other departments	8,757	(78.4)
Marital status	Married	7,750	(69.3)
	Others (Single, divorced, or widowed)	3,433	(30.7)
No. of children	0	4,307	(38.5)
	1	4,783	(42.8)
	≧2	2093	(18.7)
Living with the parents	Yes	5,942	(53.1)
	No	5,241	(46.9)
Attitude of your family	Strongly support	3,966	(35.5)
	Support	4,389	(39.2)
	A bit not support	2,481	(22.2)
	Not support at all	347	(3.1)
Experience			
Participated in similar public health crisis	Yes	742	(6.6)
	No	10,441	(93.4)
Friends or family had participated in similar public health crisis	Yes	2,884	(25.8)
	No	8,299	(74.2)
With isolated colleagues	Yes	564	(5.0)
	No	10,619	(95.0)
Close contacts with COVID−19 patients	Yes	892	(8.0)
	No	10,291	(92.0)
Training condition			
Received training for COVID−19	Frequently	8,985	(80.3)
	Sometimes	1970	(17.6)
	No	228	(2.0)
Understanding of related knowledge	Very good understanding	1594	(14.3)
	Good understanding	6,741	(60.3)
	Partly understanding	2,786	(24.9)
	Not understanding	62	(0.6)
Time spent on learning related knowledge	<1h per day	5,244	(46.89)
	1−3h per day	5,318	(47.55)
	>3h per day	621	(5.55)
Personal health			
Health status	Energetic and spirited	2072	(18.5)
	As stable as before	4,909	(43.9)
	Felt depressed	2,709	(24.2)
	Unhappy for a long time	349	(3.1)
	Sleep disorders	955	(8.5)
	Others	189	(1.7)
Stress level^a^	Range: 0 –100	57.84	23.11
Anxiety level^a^	Range: 0 –100	52.80	23.78
Perceptions			
Perceived greatest benefit	Honorary certification	63	(0.6)
	Economic compensation	87	(0.8)
	To promote professional skills	1,363	(12.2)
	To get promoted	128	(1.1)
	To realize personal value	6,323	(56.5)
	To contribute to the society	3,027	(27.1)
	Others	192	(1.7)
Perceived worst outcome	Being infected	5,775	(51.6)
	Psychological distress	196	(1.8)
	Isolated from the family	2045	(18.3)
	Unable to take care of family	2,747	(24.6)
	Others	420	(3.8)
The key facilitator for the willingness decision	Promise from administrators	603	(5.4)
	Family support	2,675	(23.9)
	Encouragement of administrators	839	(7.5)
	The impact of role models	3,757	(33.6)
	Professional ability	2,921	(26.1)
	Others	388	(3.5)
The key barrier for the willingness decision	Family's disagreement	5,101	(45.6)
	The rapid epidemic	1,197	(10.7)
	Experience of front‐line nurses	604	(5.4)
	Uncertainty in working time	513	(4.6)
	Lack of protective equipment	2,755	(24.6)
	Others	1,013	(9.1)
Bought insurance	Yes	2,500	(22.4)
	No	8,683	(77.6)
Willingness to help Hubei	Yes	9,331	(83.4)
	No	1852	(16.6)

^a^ variables presented as mean, standard deviation.

Most nurses had stable health as before (43.9%) or were energetic and spirited (18.5%). The mean level of stress was 57.84 (*SD* 23.11, range 0–100) in the VAS measurement. The mean anxiety level was 52.80 (*SD* 23.78, range 0–100). Only 6.6% of the nurses experienced a similar kind of public health crisis and 8.0% of them had close contact with suspected or diagnosed patients. Most nurses received related training for the COVID‐19 (98.0%), spent 1–3 hr per day learning related knowledge (47.55%) and had a good understanding of related knowledge (14.3% for very good, 60.3% for good). The attitudes of nurses’ families were generally supportive, with 35.5% ‘strongly support’ and 39.2% being ‘support’. Only 22.4% of the nurses had bought insurance for themselves.

### 3.2 Willingness and characteristics

As to their willingness, 83.4% of the nurses were willing to volunteer to practice in Hubei in the epidemic. The chi‐square tests and *t‐*tests examined the association between characteristics of nurses and their willingness choices. Except for gender, age and education levels, all the other demographic characteristics (such as the location, professional qualification, working department, political party membership, marital status), personal health, previous experience, training condition, and personal perceptions revealed significant associations (all *p* values < .05) with the willingness of nurses to practice in Hubei (Table 2).

**Table 2:** Comparison of characteristics between the nurses with different willingness (*N* = 11,183)

Variables	Willingness to aid Hubei				Chi square	*p* value
	Willing		Not willing			
	*N*	%	*N*	%		
Sociodemographic characteristics						
Location						
Hunan	1893	(20.3)	449	(24.2)	97.062	<.001
Chongqing	4,693	(50.3)	1,065	(57.5)		
Xinjiang	2,745	(29.4)	338	(18.3)		
Gender						
Male	320	(3.4)	52	(2.8)	1.857	.173
Female	9,011	(96.6)	1,800	(97.2)		
Age						
20–29 years	4,628	(49.6)	888	(47.9)	5.951	.051
30–39 years	3,681	(39.4)	783	(42.3)		
≧40 years	1,022	(11.0)	181	(9.8)		
Professional qualification						
Primary (registered nurses)	6,858	(73.5)	1,400	(75.6)	22.915	<.001
Middle (nurses in charge)	2037	(21.8)	411	(22.2)		
Senior (chief nurses)	436	(4.7)	41	(2.2)		
Political party membership						
No	8,119	(87.0)	1,690	(91.3)	26.252	<.001
The Communist Party of China	1,114	(11.9)	146	(7.9)		
Other democratic parties	98	(1.1)	16	(0.9)		
Education background						
Associate degree	4,076	(43.7)	787	(42.5)	7.329	.062
Bachelor degree	5,173	(55.4)	1,051	(56.7)		
Master degree or above	81	(0.9)	12	(0.6)		
Years of working						
< 3 years	1606	(17.2)	278	(15.0)	15.185	.002
3–5 years	1503	(16.1)	325	(17.5)		
5–10 years	3,080	(33.0)	675	(36.4)		
>10 years	3,142	(33.7)	574	(31.0)		
Working departments						
Emergency department	608	(6.5)	98	(5.3)	19.909	<.001
Infectious departments	327	(3.5)	78	(4.2)		
Critical care units	1,136	(12.2)	170	(9.2)		
Other departments	7,255	(77.8)	1502	(81.3)		
Marital status						
Married	6,363	(68.2)	1,387	(74.9)	32.606	<.001
Others	2,968	(31.8)	465	(25.1)		
No. of children						
0	3,694	(39.6)	613	(33.1)	31.064	<.001
1	3,948	(42.3)	835	(45.1)		
≧2	1689	(18.1)	404	(21.8)		
Living with the parents						
Yes	4,819	(51.6)	1,123	(60.6)	50.177	<.001
No	4,512	(48.4)	729	(39.4)		
Attitude of your family						
Strongly support	3,839	(41.1)	127	(6.9)	2,218.103	<.001
Support	3,900	(41.8)	489	(26.4)		
A bit not support	1,463	(15.7)	1,018	(55.0)		
Not support at all	129	(1.4)	218	(11.8)		
Experience						
Participated in similar public health crisis						
Yes	652	(7.0)	90	(4.9)	11.297	.001
No	8,679	(93.0)	1762	(95.1)		
Friends or family had participated in similar public health crisis						
Yes	2,526	(27.1)	358	(19.3)	48.379	<.001
No	6,805	(72.9)	1,494	(80.7)		
With isolated colleagues						
Yes	447	(4.8)	117	(6.3)	7.524	.008
No	8,884	(95.2)	1735	(93.7)		
Close contacts with patients						
Yes	722	(7.7)	170	(9.2)	4.375	.036
No	8,609	(92.3)	1682	(90.8)		
Training condition						
Received training for COVID−19						
Frequently	7,650	(82.0)	1,335	(72.1)	102.250	<.001
Sometimes	1,520	(16.3)	450	(24.3)		
No	161	(1.7)	67	(3.6)		
Understanding of related knowledge						
Very good understanding	1,445	(15.5)	149	(8.0)	247.717	<.001
Good understanding	5,759	(61.7)	982	(53.0)		
Partly understanding	2090	(22.4)	696	(37.6)		
Not understanding	37	(0.4)	25	(1.3)		
Time spent on learning related knowledge						
<1h per day	4,156	(44.5)	1,088	(58.7)	135.836	<.001
1−3h per day	4,619	(49.5)	699	(37.7)		
>3h per day	556	(6.0)	65	(3.5)		
Health						
Health status						
Energetic and spirited	1955	(21.0)	117	(6.3)	363.761	<.001
Stable	4,185	(44.9)	724	(39.1)		
Felt depressed	2047	(21.9)	662	(35.7)		
Unhappy for a long time	260	(2.8)	89	(4.8)		
Sleep disorders	740	(7.9)	215	(11.6)		
Others	144	(1.5)	45	(2.4)		
Stress level^a^	56.01	23.10	68.81	19.92	t = 16.57	<.001
Anxiety level^a^	50.75	23.57	65.05	21.22	t = 16.30	<.001
Perceptions						
Perceived greatest benefit						
Honorary certification	28	(0.3)	35	(1.9)	308.670	<.001
Economic compensation	51	(0.5)	36	(1.9)		
To promote professional skills	1,123	(12.0)	240	(13.0)		
To get promoted	64	(0.7)	64	(3.5)		
To realize personal value	5,399	(57.9)	924	(49.9)		
To contribute to the society	2,551	(27.3)	476	(25.7)		
Others	115	(1.2)	77	(4.2)		
Perceived worst outcome						
Being infected	4,820	(51.7)	955	(51.6)	72.002	<.001
Psychological distress	168	(1.8)	28	(1.5)		
Isolated from the family	1,800	(19.3)	245	(13.2)		
Unable to take care family	2,176	(23.3)	571	(30.8)		
Others	367	(3.9)	53	(2.9)		
The key facilitator for the willingness decision						
Promise from administrators	423	(4.5)	180	(9.7)	307.493	<.001
Family support	2,186	(23.4)	489	(26.4)		
Encouragement of administrators	701	(7.5)	138	(7.5)		
The impact of role models	3,348	(35.9)	409	(22.1)		
Professional ability	2,435	(26.1)	486	(26.2)		
Others	238	(2.6)	150	(8.1)		
The key barrier for the willingness decision						
Family's disagreement	4,153	(44.5)	948		62.847	<.001
The rapid epidemic	956	(10.2)	241	(13.0)		
Experience of front‐line nurses	507	(5.4)	97	(5.2)		
Uncertainty in working time	438	(4.7)	75	(4.0)		
Lack of protective equipment	2,385	(25.6)	370	(20.0)		
Others	892	(9.6)	121	(6.5)		
Bought insurance						
Yes	2,162	(23.2)	338	(18.3)	21.546	<.001
No	7,169	(76.8)	1514	(81.7)		

^a^ presented as mean, standard deviation, and examined by independent *t* tests.

### 3.3 Perceptions of nurses towards volunteering to help Hubei

‘To realize personal value’ (56.5%) ranked No.1 in nurses’ perception of the greatest benefit for being a volunteer to practice in Hubei, followed by ‘to contribute to the society’ (27.1%) and ‘to promote professional skills’ (12.2%). Few nurses considered personal profits in decision‐making, for example, chances for promotion (1.1%) or honorary certification (0.6%). As for the worst outcome of being a volunteer, the participants answered ‘being infected’ (51.6%) the most, followed by ‘unable to take care of the family’ (24.6%) and ‘short supply of protective equipment’ (13.3%). The top three facilitators for the willingness decision of nurses were ‘the impact of role models’ (33.6%), ‘professional ability’ (26.1%), and ‘family support’ (23.9%). The three key barriers included ‘family disagreement’ (45.6%), ‘lack of protective equipment’ (24.6%), and ‘the rapid epidemic’ (10.7%).

### 3.4 Factors associated with the willingness of nurses

The binary logistic regression analysis findings were summarized in Table 3. The final regression model included the variables of location, age, professional qualification, working department, political party membership, marital status, attitude of families, training, time spent on learning related knowledge, health condition, and anxiety levels (all *p* values < .05). These variables could explain 31.1% of the variations in the willingness decision of nurses (*p* < .001).

**Table 3:** Logistic regression model for the willingness of nurses to help Hubei combat COVID‐19 (*N* = 11,183)

Variables	B	S.E.	Wald	*df*	*p*	Odds ratio	95% CI	
Constant	−0.627	0.644	0.946	1	0.331	0.534	Lower	Upper
Location								
Hunan	−0.302	0.218	1.929	1	0.165	0.739	0.482	1.132
Chongqing	−0.409	0.100	16.558	1	<0.001	0.664	0.546	0.809
Gender								
Females	0.224	0.268	0.697	1	0.404	1.251	0.739	2.118
Age								
30–39 years	−0.203	0.141	2.059	1	0.151	0.816	0.619	1.077
≧40 years	−0.734	0.224	10.757	1	0.001	0.480	0.309	0.744
Professional qualification								
Middle (nurses in charge)	0.065	0.137	0.222	1	0.637	1.067	0.815	1.396
Senior (chief nurses)	0.851	0.346	6.041	1	0.014	2.342	1.188	4.616
Political party Membership								
The Communist Party of China	0.357	0.166	4.644	1	0.031	1.429	1.033	1.978
Other democratic parties	0.124	0.477	0.068	1	0.795	1.132	0.444	2.886
Education								
Bachelor degree	0.178	0.095	3.534	1	0.060	1.195	0.992	1.439
Master degree or above	0.645	0.692	0.869	1	0.351	1.907	0.491	7.409
Years of working								
3–5 years	−0.168	0.223	0.566	1	0.452	0.845	0.546	1.309
5–10 years	−0.190	0.196	0.935	1	0.334	0.827	0.563	1.215
>10 years	−0.093	0.140	0.440	1	0.507	0.912	0.693	1.199
Working department								
Emergency department	−0.018	0.176	0.011	1	0.918	0.982	0.696	1.386
Infectious departments	−0.714	0.212	11.340	1	0.001	0.490	0.323	0.742
Critical care units	0.437	0.152	8.232	1	0.004	1.549	1.149	2.088
Marital status								
Married	−0.392	0.174	5.091	1	0.024	0.675	0.480	0.950
No. of children								
1	−0.059	0.162	0.133	1	0.715	0.942	0.685	1.296
≧2	−0.152	0.186	0.671	1	0.413	0.859	0.597	1.236
Living condition								
Living with parents	−0.161	0.093	3.015	1	0.083	0.851	0.710	1.021
Attitude of family								
Strongly support	3.420	0.227	227.573	1	<0.001	30.558	19.596	47.651
Support	2.421	0.203	142.625	1	<0.001	11.259	7.567	16.752
A bit not support	0.947	0.199	22.618	1	<0.001	2.577	1.745	3.807
Participated in public health crisis	0.156	0.191	0.666	1	0.414	1.169	0.804	1.699
Friends/ family participated in public health crisis	0.199	0.112	3.199	1	0.074	1.221	0.981	1.519
With isolated colleagues	−0.294	0.187	2.473	1	0.116	0.745	0.517	1.075
Had close contact with patients	−0.170	0.173	0.965	1	0.326	0.844	0.601	1.184
Bought Insurance	0.147	0.108	1.843	1	0.175	1.159	0.937	1.433
Received training								
Frequently	0.627	0.243	6.667	1	0.010	1.873	1.163	3.015
Sometimes	0.454	0.246	3.401	1	0.065	1.575	0.972	2.553
Understanding of related knowledge								
Very good understanding	0.294	0.465	0.399	1	0.528	1.342	0.539	3.340
Good understanding	0.534	0.445	1.439	1	0.230	1.706	0.713	4.081
Partly understanding	0.475	0.439	1.171	1	0.279	1.608	0.680	3.800
Time spent on learning daily								
1−3h	0.230	0.093	6.124	1	0.013	1.259	1.049	1.511
>3h	0.258	0.238	1.176	1	0.278	1.294	0.812	2.062
Health status								
Energetic and spirited	1.017	0.299	11.559	1	0.001	2.766	1.539	4.971
Stable	0.707	0.269	6.913	1	0.009	2.027	1.197	3.433
Depressed	0.574	0.271	4.470	1	0.034	1.775	1.043	3.021
Unhappy for a long time	0.565	0.331	2.902	1	0.088	1.759	0.919	3.367
Sleep disorders	0.665	0.290	5.248	1	0.022	1.945	1.101	3.435
Stress level	−0.036	0.038	0.885	1	0.347	0.965	0.895	1.040
Anxiety level	−0.142	0.037	15.026	1	<0.001	0.867	0.807	0.932

Note

R^2^ = 0.311, *p* < .001. Reference for the two‐or‐more categorical variables. Location: Xinjiang as the reference variable; Age: 20–29 years as the reference variable; Professional qualification: primary (registered nurses) as the reference variable; Political party membership: no membership as the reference variable; Education: associate degree as the reference variable; Years of working: <3 years as the reference variable; Working departments: Other departments as the reference variable. No. of child: with no child as the reference variable; Attitude of family: not support at all as the reference variable; Training: received no training as the reference variable;; Understanding of related knowledge: not understanding as the reference variable; Time spent on learning related knowledge:<1 hr per day as the reference variable; Health status: Others as the reference variable.

## 4 DISCUSSION

Nurses play critical roles in the epidemic caused by infectious diseases. Given the large number of COVID‐19 patients and the shortage of nursing workforce in Hubei, nurses from other provinces were urgently needed to practice in Hubei combating this epidemic. Therefore, understanding the willingness of nurses was critical in addressing the shortage of nursing workforce. Identifying the associated factors would contribute to engaging nurses to participate in such kind of epidemic.

The demographic characteristics of the current participants were consistent with those in the National Nurses Survey of China (NNSC) (China Social Welfare Foundation, 2017). Although 43.9% of the nurses reported stable health conditions as before, they showed a middle level of stress (57.84 out of 100) and anxiety (52.80 out of 100). Similar to other studies, the outbreaks of COVID‐19 might impose additional stress on nurses (Oh et al., 2017). Some nurses felt depressed (24.2%), unhappy (3.1%), or had sleep disorders (8.5%). This finding was consistent with the results of the NNSC, where 86% of the nurses expressed the need for psychological support to release their stress from daily work (China Social Welfare Foundation, 2017). This finding indicates that professional support should be provided to promote the psychological health of nurses not only when an infectious disease occurs, but also as a routine practice.

### 4.1 Willingness of nurses to volunteer to help Hubei

Our study revealed that a large proportion of nurses (83.4%) were willing to practice in Hubei, which was much higher than that in previous outbreaks of infectious diseases (Khalid et al., 2016; Lee et al., 2005; Turtle et al., 2015; Wong et al., 2010).

The possible reasons for such a strong willingness might be related to the current social environment in China. On the one hand, most Chinese nurses were eager to be respected as professionals and to increase their social status (China Social Welfare Foundation, 2017). The professionalism of nurses in caring for the COVID‐19 patients would help nurses realize their professional value and gain further social recognition. In the current study, the nurses perceived ‘to realize personal value’ (56.5%), ‘to contribute to the society’ (27.1%) and ‘to promote professional skills’ (12.2%) as the top benefits of being a volunteer. Unlike the findings in the Middle East (Khalid et al., 2016), relatively fewer nurses in China considered personal benefits in their decision making, for example, promotion or economic compensation. These internal motivations would definitely enable nurses to be one of the frontline fighters. On the other hand, the experience of frontline nurses engaged the participants to make a willing decision. ‘The experience of role models’ was the top facilitator (33.6%) for the decision of current nurses. Since the end of January 2020, the Chinese government has launched series of national activities to publicly acknowledge the contributions of frontline healthcare professionals (including the first group of volunteers) by television, radio, Internet, newspapers, and other media (NHC, 2020f). The professional performance and contributions of frontline nurses in this epidemic were broadly recognized by the entire society. Moreover, the Chinese culture cherishes the interests of the whole nation and respect the spirit of sacrifice (Lee et al., 2005). Our finding also confirmed that the members of the Communist Party of China were more likely to make a willing choice. The Communist beliefs and their commitment to the call of the central government engaged these nurses to volunteer. This kind of social and cultural environment would be another reason for the choices of nurses to volunteer to help Hubei.

Similar with other studies in Asia (Lee et al., 2005, Oh et al., 2015), family support is another key facilitator in the current study. As high as 72.7% of the current participants reported the supportive attitude of their family towards them to be a volunteer. The regression analyses also confirmed that nurses with support from their families were more likely to make a willing decision.

In addition to Chinese‐specific factors, professionalism also engaged the willingness of nurses to practice in Hubei. In the current study, 26.1% of the nurses regarded ‘professional ability’ as the key facilitator for their willingness decision. Moreover, the logistic regression findings indicated those who had senior professional qualification, received frequent training on COVID‐19 and spent 1–3 hr per day learning about related knowledge were more likely to make a willing decision. Similar with the findings in previous studies (Khalid et al., 2016; Lee et al., 2005; Turtle et al., 2015), professional knowledge and related training would increase the confidence of nurses and prepare them better to fight against the outbreak. However, professional knowledge would also compel nurses to be greatly worried about the ‘lack of protective equipment’ and ‘the rapid epidemic’ in the COVID‐19 epidemic.

### 4.2 Factors associated with the willingness decision of nurses

The logistic regression indicated that compared with the 20–29‐year group and the unmarried group, nurses who were over 40 years and married were less likely to make a willing choice. This result might be related to the key family roles in China. Married and older nurses might have greater family commitment towards caring for the parents and children, leading to their unwillingness decision (Lee et al., 2005).

An interesting finding was that nurses from Chongqing were less likely to help Hubei, compared with those in Xinjiang. Given the adjacent location, convenient transportation and a huge number of migrant populations with Hubei, Chongqing was estimated to be another epicentre of the COVID‐19 epidemic (Caixin Health, 2020). On 27 January 2020, Chongqing reported 132 COVID‐19 patients, ranking No.1 amongst all the cities outside Hubei (Chongqing Health Commission, 2020). The rapid growth of the epidemic in Chongqing might impose essential stress on local nurses, thus diminishing their willingness to serve in Hubei.

Another interesting phenomenon was identified in working departments. Nurses working in infectious diseases departments were less willing to practice in Hubei. Given the rapid spread of the COVID‐19 throughout China, nurses of infectious diseases departments might have already been fully occupied with screening suspected patients and caring for infected patients locally. They might not have extra energy to help Hubei. By contrast, critical care nurses were more willing to be deployed to Hubei. As reported by the WHO (2020), the rate of severe cases caused by the COVID‐19 was lower than SARS‐CoV. The COVID‐19‐related occupation rate in critical care departments of the study provinces was much lower than that in Hubei. Given their outstanding professional ability, critical care nurses would be confident in joining the combat with COVID‐19.

In the current study, health condition of nurses was significantly associated with their willingness decision, which was consistent with previous studies (Damery et al., 2009; Lee et al., 2005; Wong et al., 2010). Nurses with good health (energetic and spirited, as stable as before) might be greatly confident with their personal immunity and were more likely to volunteer, whereas those with high levels of anxiety were less likely to make a willing decision.

The current study indicated that nurses with the following characteristics were more likely to volunteer to practice in Hubei: those who were younger, unmarried, member of the Communist Party of China, with senior professional qualification, working in critical care departments, with support from the family, with adequate training and learning, with good health status and lower level of anxiety. Adequate training and psychological support might be feasible interventions to maintain the willingness of nurses to serve in the frontlines. The public acknowledgement of their professional contributions and support from their family might also help to engage nurses to volunteer.

### 4.3 Limitations

The current study indicated the feasibility and high efficiency of an online survey among nurses in the outbreak of an infectious disease. There are also some limitations in this study. Firstly, it was an online survey using convenient sampling. Secondly, the willingness, health status, stress, and anxiety levels of nurses were measured by single questions. To facilitate the online survey and response rate, only multiple‐choice questions were designed. In‐depth reasons for the willingness choices of nurses were not explored. Future studies could employ the systematic sampling methods. Qualitative studies were also suggested to provide more comprehensive understanding about the willingness of nurses during the outbreak of an infectious disease.

## 5 CONCLUSION

The current study revealed that a high proportion of nurses in China were willing to practice in Hubei during the outbreak of COVID‐19. Adequate training and psychological support would facilitate nurses to volunteer during the epidemic of an infectious disease.

## CONFLICT OF INTEREST

The authors declare no conflict of interest.

## Author contributions

XQG: conceptualization, data curation, investigation, formal analysis, resources, writing – review & editing. ZYS: conceptualization, data curation, investigation, formal analysis, resources, writing‐review & editing. SYC: conceptualization, methodology, supervision, validation, writing‐review & editing. XC: formal analysis, project administration, writing‐original draft, writing‐review & editing. QW: conceptualization, methodology, formal analysis, writing‐original draft, writing‐review & editing.
